# Supplementary material for: Genome sequence of segmented filamentous bacteria present in the human intestine
Source: Commun Biol. 2020 Sep 4;3:485. doi: 10.1038/s42003-020-01214-7 (PMC7474095; doi:10.1038/s42003-020-01214-7)
Supplement: Supplementary file 1 — Supplementary Information [file 42003_2020_1214_MOESM1_ESM.pdf]

Supplementary figures

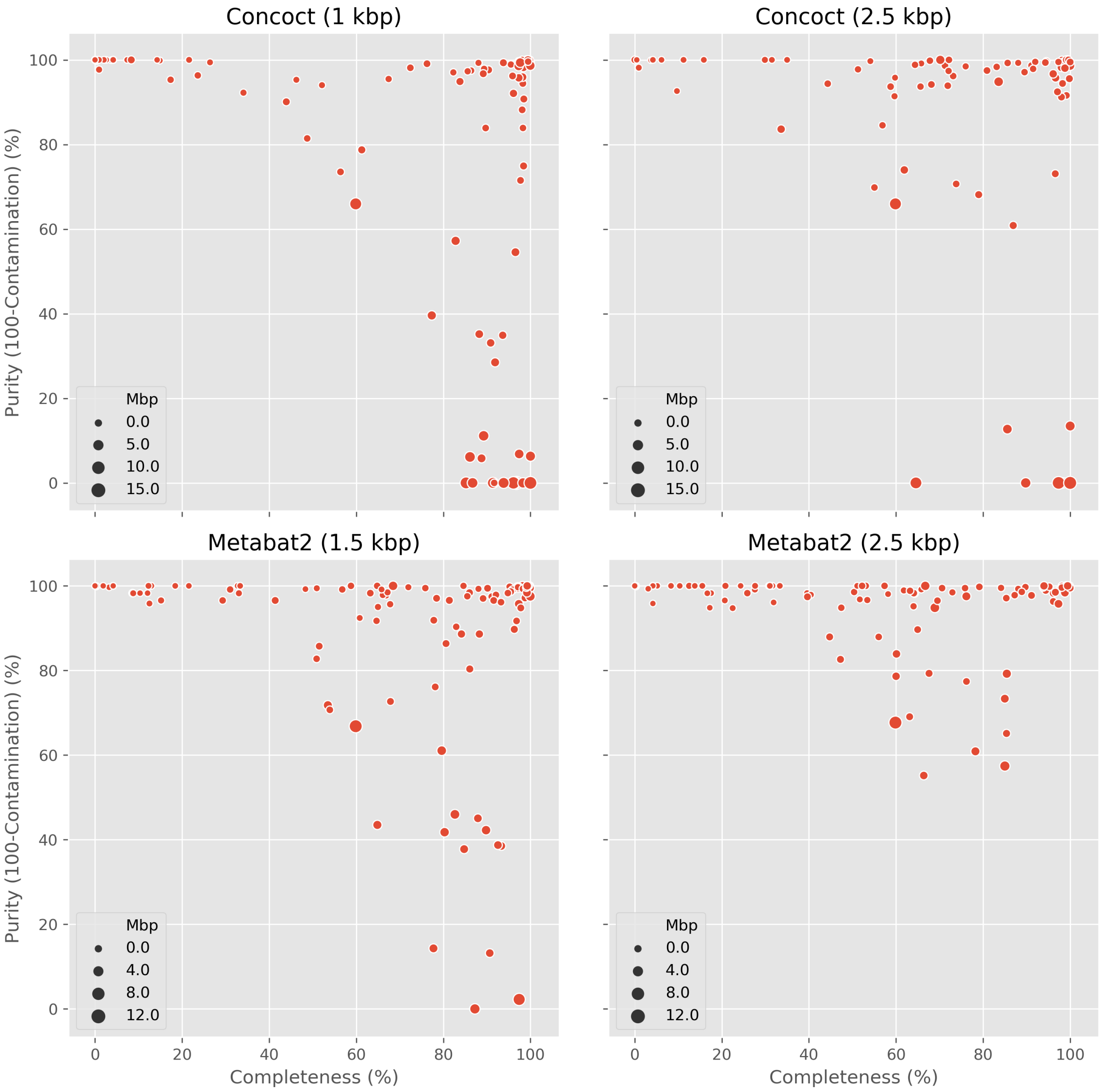

Supplementary Figure 1. Metagenomic bins obtained with CONCOCT and Metabat2 using different cutoffs on minimum contig length. Each circle represents one bin, with its position along the x- and y-axis indicating estimated completeness and purity, respectively.

>sfb.merged_k141_89555_16S-rRNA

```
GATAAGAGTTTGATCCTGGCTCAGGACGAACGCTGGCGGCGTGCCTAACACATGCAAGTTGAACG
GAGTTATGTGGATTACTTTAGGGTAATTTATGTAATTTAGTGGCGAACGGGTGAGTAACACGTAG
ATAATCTGTCCTATATTGGGGGATAGGCCGATGAAAGTTGGATTAATACCGCATATAGCTAATAT
ATTGCATGATATATTAGTGAAAGGGAGAAATTTTGATATAGGAGGAGTCTGCGACACATTAGCTA
GTAGGTAAGGTAAAGGCTTACCTAGGCGACGATGTGTAGCTGGTCTGAGAGGATGGACGGCCACA
ATGGAACTGAGACACGGTCCATACTCCTACGGGAGGCAGCAGTGGGGAATATTGCACAATGGGGG
GAACCCTGATGCAGCAACGCCGCGTGAGTGAAGAAGGTTTTCGGATTGTAAAGCTCTGTTAGCAG
GGAAGAGGAAGGACGGTACCTGCAGAGGAAGCCACGGCTAACTACGTGCCAGCAGCCGCGGTAAT
ACGTAGGTGGCAAGCGTTGTTCGGAATAACTGGGCGTAAAGGATGCGTAGGCGGTTATATAAGTT
ATGTTGTTAAATATACAGGCTTAACCTGTAGAAAGCGGATAAAACTGTATGACTTGAGTGCAGGA
GAGGTAAGTGGAATTTCTAGTGTAGCGGTGAAATGCGTAGAGATTAGGAAGAACACCAGTGGCGA
AGGCGACTTACTGGACTGTAACTGACGCTGAGGCATGAGAGCATGGGGAGCAAACAGGATTAGAT
ACCCTGGTAGTCCATGCTGTAAACGATGGGTACTAGGTGTGGGTTGTGAATAGCAATCTGTGCCG
TCGCAAACGCAATAAGTACCCCGCCTGAGGAGTACGATCGCAAGATTAAAACTCAAAGGAATTGA
CGGGGACCCGCACAAGCAGCGGAGCATGTGGTTTAATTCGAAGCAACGCGAAGAACCTTACCTAG
ACTTGACATACCTTGAATTACCTTGTAATGAGGGAAGCTCGCAAGAGCAAGGATACAGGTGGTGC
ATGGTTGTCGTCAGCTCGTGTCGTGAGATGTTGGGTTAAGTCCCGCAACGAGCGCAACCCTTGTT
GTTAATTGCTAGCAGGTGAAGCTGAGCACTTTAGCGAGACAGCCTAGGTTAACTAGGAGGAAGGT
GGGGATGACGTCAAATCATCATGCCCCTTACGTCTAGGGCTACACACGTGCTACAATGGTGAGAA
CAGAGAGAAGCAAGCTAGTGATAGTGAGCAAACCTTATAAAACTCATCTCAGTTCGGATTGCAGG
CTGAAACTCGCCTGTATGAAGATGGAGTTGCTAGTAATCGCGAATCAGAATGTCGCGGTGAATAC
GTTCCCGGGTCTTGTACACACCGCCCGTCACACCATGAGAGTTGGCAACACCCGAAGCCTGTGAG
CTAACCGAAAGGAGGCAGCAGTCTAAGGTGGGGTTAATGATTGGGGTGAAGTCGTAACAAGGTAG
CCGTAGGAGAACCTGCGGCTGGATCACCTCCTTTC
```

Published SFB probes / PCR primers

SFBf1	5'-GGAGTCTGCGGCACATTAG-3'	Johnsson, 2013
779F	5'-TGTGGGTTGTGAATAACAAT-3'	Urdaci, 2001
1008R	5′-GCGAGCTTCCCTCATTACAAGG-3'	Snel, 1994
1008R	5'-GCGGGCTTCCCTCATTACAAGG-3'	*Yin, 2013
1380R	5'-GGTTAGCCCACAGGTTTCGG-3'	Urdaci, 2001
1380R	5'-GGTTAGCCCACAGGCTTCGG-3'	*Yin, 2013
SFBr1	5'-CACCTTAGACTGCTGCCTC-3'	Johnsson, 2013

*Sequences as given in Supplementary Table S2 in Yin et al.

Supplementary Figure 2. The 16S rRNA gene sequence of SFB-human-IMAG and published probes/primers used for the identification of SFB. Nucleotides in red indicate mismatches relative to primers/probes. The Johnson and Yin references are given in the main article, Snel refers to Snel et al. System. Appl. Microbiol. 17, 172-179 (1994) and Urdaci to Urdaci et al. Res Microbiol 152: 67–73 (2001).

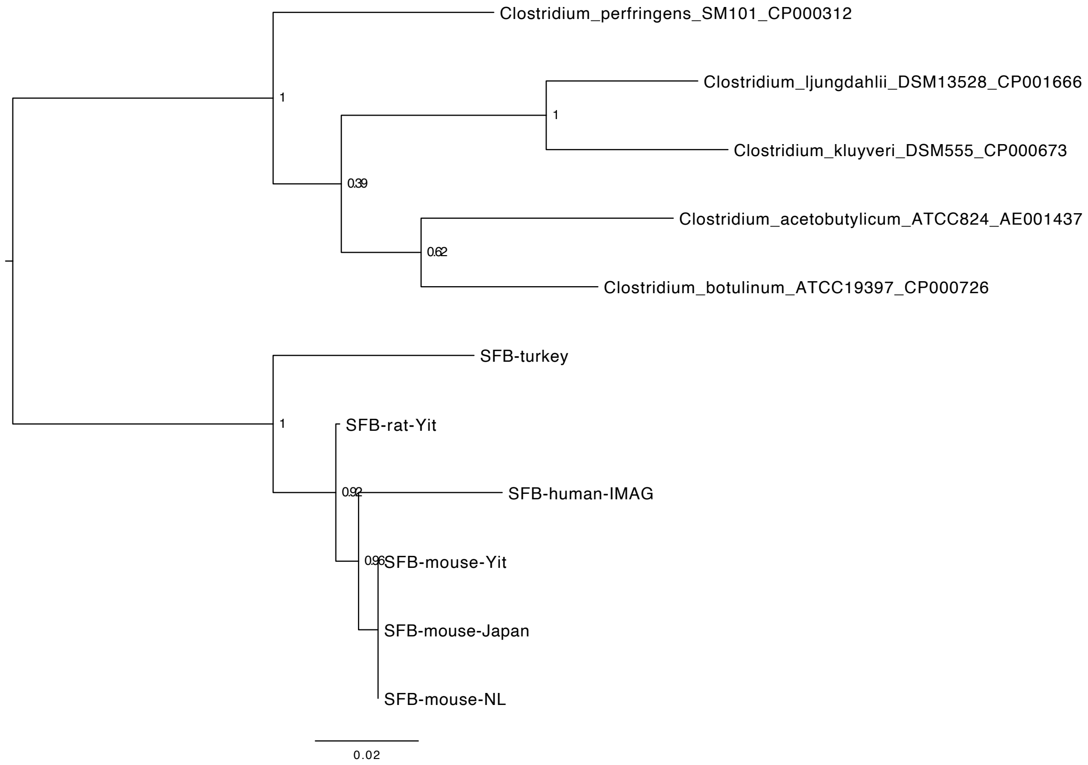

Supplementary Figure 3. Phylogenetic tree based on 16S rRNA gene sequences from sequenced SFB genomes. The 16S rRNA gene sequences from five *Clostridium* genomes served as outgroup for rooting the tree.

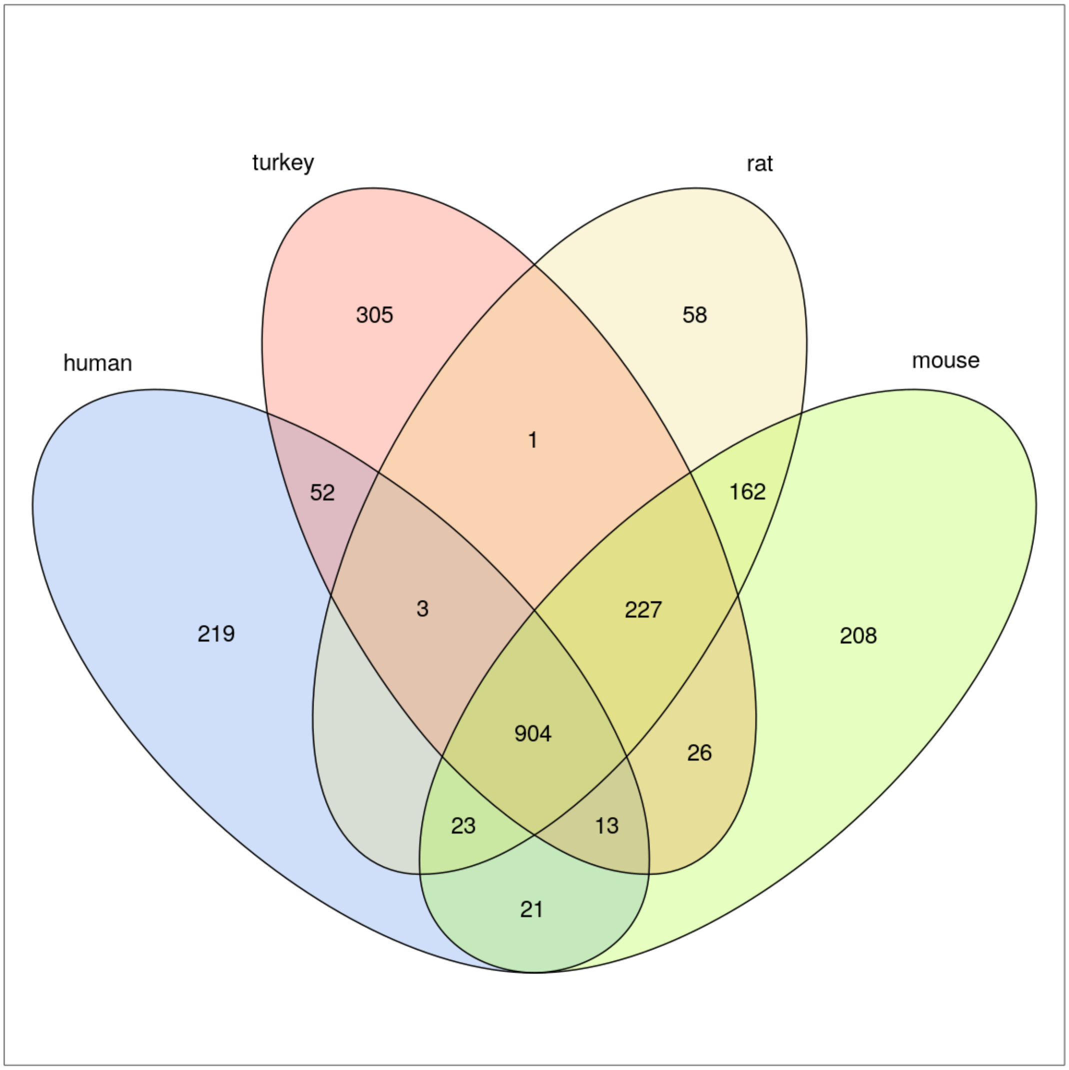

Supplementary Figure 4. Venn diagram depicting the overlap in gene clusters for the SFB lineages from different hosts. The mouse lineage represents the union of SFB-mouse-Yit, SFB-mouse-Japan and SFB-mouse-NL.

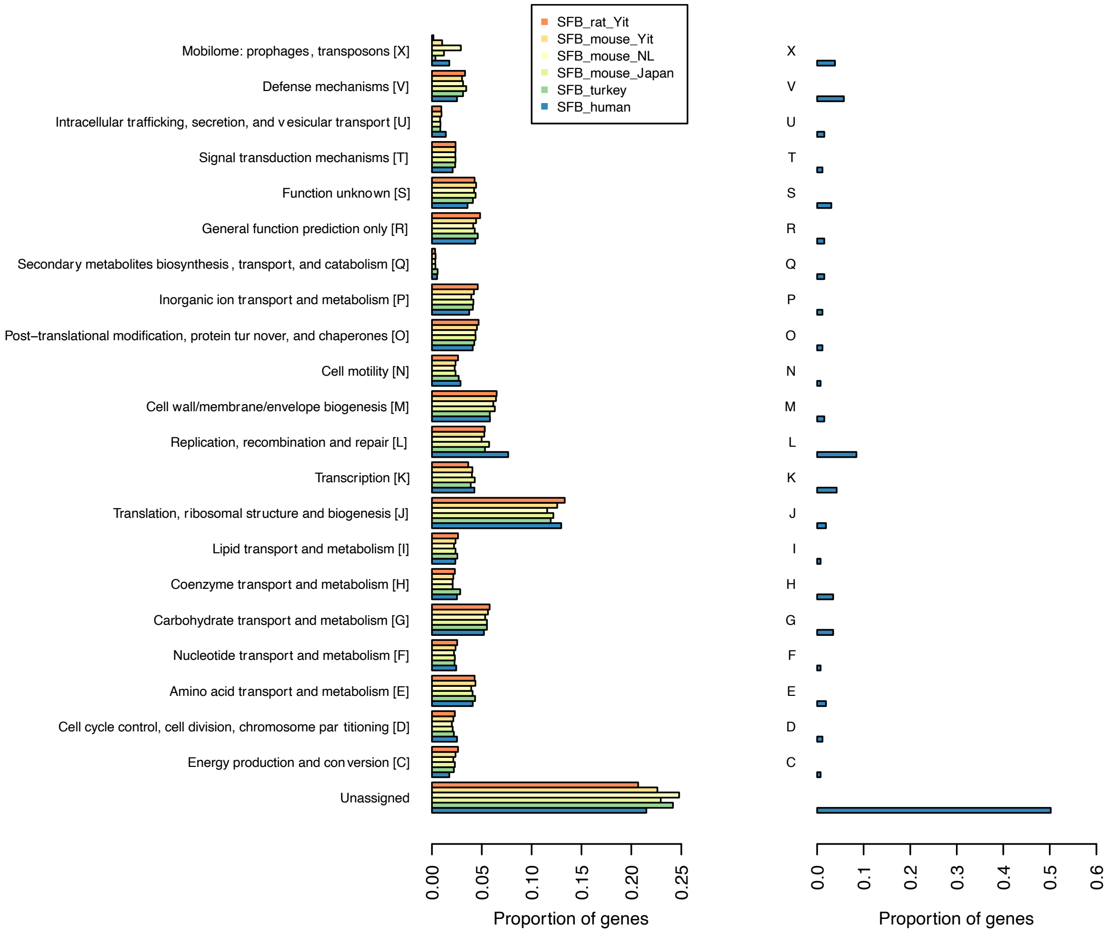

Supplementary Figure 5. Proportion of genes assigned to different COG functional categories. (A) Calculated based on all genes of the different SFB genomes. (B) Calculated based on the SFB-human-IMAG genes that belong to protein clusters that were not found in any of the rodent SFB. COG categories V and X were enriched in these protein clusters relative to the overall genome, while categories J, M, N, O, P and R were depleted (Fisher's Exact Test, False Discovery Rate adjusted *P*-value < 0.05).

```
SFBM_0962            MKLTFNSNAMKTFMTYKKTISDHSKSIKNISTGKQINSAKDNPSRISKLANFEKEIRGYQ   60
00941                MKLNFNSNAVKTLRAYKKNILDHSKNINNISTGKKINSSKDNPNHISKLGNFEREIRGYQ   60
00379                MIINHNLNVMNAHRNMSNSVKSQGKSMEKLSSGLRIGRAGDDAAGLAISEKMRTQIRGLD   60
SFBM_0642            MIINHNINAMNAHRNMGSTTIAQGKAMEKLNSGLRINRAADDAAGFAISEKMRSQIRGLN   60
00424                MIINHNLNAMNAHRNMTGTTTAQGKAMEKLSSGLRINRAGDDAAGLAISEKMRSQIRGLD   60
SFBM_0583            MIINHNMNAMNAHRNMGSTTVSQGKSMEKLSSGLRINRAGDDAAGLAISEKMRSQIRGLN   60
SFBM_0584            MIINHNMNAMNAHRNMGSTTIAQGKAMEKLNSGLRINRAADDAAGLAISEKMRSQIRGLN   60
                     * :..* *.:::       .    :.* ::::.:* :*. : *:    ::    ::. :*** :

SFBM_0962            SSRRNIQDTVSMIQSADSVMGSLNDRVTRLKEITISLGNGSIQGDEKDIIQNEVNSILEG   120
00941                ASRRNIQDTVSMIQAADGVLDSVGNRITRIRELSVELGNASIGEDEKNILGNEINTLLEG   120
00379                QASRNAQDGIPMVQTAEGALTETHAITQRMRELAVQSANGTYTDEDREIINQEFTQLKKE   120
SFBM_0642            QASRNTQDGISMIQTAEGALSETQAIAQRMRELAVQSANGTYTDEDRTLIDQEFKKLKSE   120
00424                QASRNAQDGISMIQTAEGALSETQAIGQRMRELAVQSANGTYTDEDRKLIDQEFNQLKSE   120
SFBM_0583            QASRNAQDGISMIQTAEGALSETQAIGQRMRELAVQSANGTYTDEDRELINQEFNQLKSE   120
SFBM_0584            QASRNAQDGISMIQTAEGALSETQAIAQRMRELAVQSANGTYTDEDRTLIDQEFNQLKSE   120
                      : ** ** : *:*:*:..: .       *::*:::. .*.:    ::: :: :*.. : .

SFBM_0962            MSYDIEHFSFNEINILGNKDVTDNSNPKLI------------------------------   150
00941                IDYEIKNFSFNGINVLGHGETNDNSNPQLV------------------------------   150
00379                IDRIADDTDFGGVKVLSGSISSKNIQPGDVKRD----NGKCTMN----FYATGFKLTKQD   172
SFBM_0642            IDRISEDTEFNGSKLINGSISGKEIKTRDKDS--VII--NGDIKNPNSINEVMKEIDPNE   176
00424                IDRIATDTEFNGNKVINGNLSSKKIVEGT------VTKGTGSMNNVTEADLEFFEGDV--   172
SFBM_0583            IDRIANDTEFNGSKVLNGDKSGEKIEFDTGSITSTAVNGGTPLDA-ANAVNVFLETDLKN   179
SFBM_0584            IDRIATDTDFSGSKVLNGEKSGEKIEFESGTVASTG---TGTLNN-TDAAAVFLKTDLEN   176
                     :.    . .*.  :::.     .:

SFBM_0962            -----------------------SVLTT--------------------------------   155
00941                -----------------------EVLTT--------------------------------   155
00379                AKDLGEGNFTFKVSVSDDSKVTIDLIDKSYFNDYKEYIIDSYTFDGTDPSSTLGSRDMIK   232
SFBM_0642            LVRLGEGTFDLIISKDRNLDVTISIVDRSHFNGWKPYVIDSIIIDNPSVIEDVE------   230
00424                ANILGEGTFTLDIKIDAGGVININLLDKSRFNGNEEYVMDSITIDDTQIKADIGSANKQG   232
SFBM_0583            VNTLGEGTFHLKLTVDG-NEAKIELTDTSKFNDNKAYVLDSIVIKDGAVGNDEH------   232
SFBM_0584            VNTLGEGTFKLNLSLDG-DDVKLELIDESKFNDNKAYVLDSTVIKDGAADNDEH------   229
                                           .:

SFBM_0962            --------------------------------------------------GNSGEFSEIP   165
00941                --------------------------------------------------SKSGEVTEIP   165
00379                IKIDKAEINMGLT-------GLKPGGVA--YFDLVNASPPRGDGVILQVEANQNQRVGFS   283
SFBM_0642            ----LAGINLRFNNIHATSSYLPAG--KEIRISFFNDSEKKSNGVDFQVGSNKGQMVGLD   284
00424                 AGFTIGGVDFVLDVE-----HLEKGKDIKLTITNTTEEKETGAGVELQIGANQNQMIGLS   287
SFBM_0583            -EITLGGADFKLAKLK---THIQDANVETVDLTLNNKVTEKTDGVELQVGANKDQMIGVS   288
SFBM_0584            -EITLGGAAFKLAQLK---DKIKDANVETVELTLNNVVKEKTNGVELQVGANKDQMISIS   285
                                                                  .:..:   .

SFBM_0962            SFNLSIENLGIDGMNV--KDDIGFNLEKIDNATSQIINARTKLGAISATFEDKIEQSESI   223
00941                LFNLSIESLGIKDFDV--NDDLSSILGKVDNAFSEVVSARSKLGAIGNTLEDKIKHSENL   223
00379                IENMSHRELGLGGINISTAENAQEAIARLDFSINAVSTQRADLGAIQNRLETIVTSTNNT   343
SFBM_0642            IENMRSRELGLGGSDLSTIENSKEAIERLDIAIFRVSEQRANLGSVQNRLEHTVSSIGNT   344
00424                MENMRSRELGLGGVNVATAESSQDAIGKLDEAISRISAQRADLGAAQNRLEHTIASTDNT   347
SFBM_0583            IGNMGSRELGLGGVNVSTAEDAKDAIGRLDEAVSRISAQRADLGAAQNRLEHTIASTDNT   348
SFBM_0584            IGNMRSRELGLGGVNVATTDDAKDAIGRLDEAVSRISAQRADLGASQNRLEHTIASTDNT   345
                       *:  ..**: . ::   :.    : ::* :   :   *:.**:    :*  :    .

SFBM_0962            EEVVTGAKSKIEDADVALEMLEFSRTLMLTEANIKNMSKTIYLPNDLIDVLGKLYK       279
00941                EEAIVGSKSKIEDSDIA---------------------------------------       240
00379                AENLQAAESRIRDVDMAKEMMNLTKLNILQQVS-----------------------       376
SFBM_0642            AENLQAAESRIRDVDMAKEMMNLTKLNILQQATQSMLAQANQSPQQVISILK----       396
00424                AENLQAAESRIRDVDMAKEMMNLTKLNVLQQASQSMLAQANQAPQQVLSILR----       399
SFBM_0583            AENLQAAESRIRDVDMAKEMMNLTKLNVLQQASQSMLAQANQAPQQVLSILR----       400
SFBM_0584            AENLQAAESRIRDVDMAKEMMNLTKLNVLQQASQAMLAQANQAPQQVLSLLK----       397
                      * : .::*:*.* *:*
```

Supplementary Figure 6. Clustal W alignment of the amino acid sequences of putative flagellins in SFB-Human-IMAG (00379, 00424, 00941) and SFM-mouse-Japan (SFBM_0583, SFBM_0584, SFBM_0642, SFBM_0962; Kuwahara et. al 2011). The yellow labeled amino acids depicts the most important positions for TLR5 signaling activity according to Song et. al 2017. Red color represents deviation from the consensus sequence.

```
00774               MNKKRIALALSAALGINTLMITVGNINGQVQIVHAVTDTRLND-SSIIDTLKVKTLKVQT      59
SFBNYU_003340       MNKKRVALALAAALGMNSLMVTVGAVGGQAFIAHAQDSKLSTDLLDKLKTLEIESLETSV      60
                    *****:****:****:*:**:*** :.**. *.**  ..  .*  . :.**::::*:...

00774               SMSSDGKDLYLSFPDRTDISNAKISSFNRQNFTERGNIDGNSGQVPATSSWITSGNYLSI      119
SFBNYU_003340       DMSTDGKAQVTFTKISPETISNVTGEFEYKPFTERSDLKETA--SKATGVWDKSTGKFNV      118
                    .**:***          :  .   ..*: : ****.::. .:    **. * .* . :.:

00774               SGFNSNAPGIYTTEVTITTNSGATETYKISLAKTPTDTLTAGKVT-VEPMSVTVEDLSLN      178
SFBNYU_003340       SGI--TAPGIYTGEVTIAYANKMTAKYTLSLVKKPSDSITFNTEVGVGT--VKLSNIEFD      174
                    **:  .****** ****:  .  * .*.:**.*.*:*::* .. . *    *.:.::.::

00774               GK-----ALDIGKKVFLLNSSDQEVSYGKVEDKAGTVKFNMSGVNM--KVGE----VYKI      227
SFBNYU_003340       NGSSKVTGFKPKKVSFSLNGGT-PVEVG--PDANGNYVLNLLDLGNKGNLKDTDKLTANF      231
                    .      .:.  *  * **..   *. *   *  *.  :*: .:.   :: :    . ::

00774               VYGVDKEDASALYKASTYLVMTRADNRGYDIVGATNVKDALSTAKAEIAKDFGVNTDSVA      287
SFBNYU_003340       SYGAN-----SEYTLSSDIVLVRKNQKITN-AVASNDKTKFPNIKQQIKTDFGLTTDPEI      285
                     **.:     : *. *: :*:.* :::  : . *:* *  : . * :* .***:.**

00774               IKETNTGYSVSVPKLNSSFDAKEETVLELPQTFGAASVLHGLPLTVTTVGNSLKVDFGDG      347
SFBNYU_003340       GT-----YVDNTHSYTISFGGK--ELLTYDQTVSTGDKLGGASLTLTTTGSQVKLSPESA      338
                     .     *  .. . . **..*   :*   **..:.. * *  **:**.*..:*:.  ..

00774               KI----TDTNNYVPYAQLSYGNNQS-----ETTLVGDYTATFNSGSTTLTTVSYAEFSNV      398
SFBNYU_003340       TLAKAAQLDDNYVPNVMLSYGINTAGNSTISAGLIGDYTLTIDKNTITCL-------DAM      391
                    .:       :**** . **** * :     .: *:**** *::..: *          . :

00774               TELKPSLTAVTNKA--------GTVSLGKKYDLKT---DDYTNSSANLDLSSFEHGCHEI      447
SFBNYU_003340       NGLTPSITTTNIANIDYSYVAFTGTGIGGNSELVTDMTKIDKNQSITLGIDKFEHGYNAI      451
                    . *.**:*:..              ..:* : :* *   .  .*.* .*.:..**** : *

00774               SIDFGVSVAADFATQVNDAAKNASSATASTITPNTEAKIKQSDDKSGDSVKTSILLAIDD      507
SFBNYU_003340       PVTFEVDAKNDKNTISA--------LTASMTGANNVIADINTASTSEEAVKASIIVSVDL      503
                     : * *..  *  *            ***    *.     :: ..* ::**:**::::*

00774               IDGVAVSSQFIQTDATKGKLLIKNGKSLFANGAPDSDIGS-KLSVSGAKS-VALSSSDTE      565
SFBNYU_003340       FDGVAVQSKFVKTSSSEGQVTIVGGSKLLNDLVGSLDYNEAVLTIDGVKATVNSTKSNDN      563
                    :*****.*:*::*.:::*:: * .*..*: : . . * ..  *::.*.*: *  :.*: :

00774               SLVYDVEFNNAVSDT--ISWELSGTSK-VKGET-AVQGKVAAANLSSVGIQN--APNTVT      619
SFBNYU_003340       DLIFDVQFNGAVPNKTDASWVLDIKGKKVEGKVDLTPGAVESVNLTSVQATNSGAENITD      623
                    .*::**:**.** :.   ** *. ..* *:*:.  . * * :.**:**   *  * * .

00774               KVKVNAQFTSDIPGLTAIGFGTQNTLEVDVNELTSTGAKSVRGSVSSGKYQGTYHAGVLV      679
SFBNYU_003340       QFKIDAKFNSTVPTGTVRVNDPVNGSDRVTFDQFKSGNNTIHNVVTSGKYQGTYSAGVIA      683
                    :.*::*:*.* :*  *.   .  *  :  . :  .:* ::::. *:******** ***:.

00774               DKVVGKITLEKPVTVTGNSVTLRFYTDFINEDDQNVNITKVNRSVIAYRRT----DGIGG      735
SFBNYU_003340       NYNPITVLLKA-DNPTTSSVRLTIDMDFFGNNDLIKNI---TGGIIYYREYKAGLGANDG      739
                    :    .: *:   . * .** * :  **:.::*   **    . .:* **.     .. .*

00774               WNITELSMEEAVNSITKTTTKTITGLERDTNYNFVVFMDVILTGGGFNGETHILQSNVVT      795
SFBNYU_003340       WEIAKGADISSSDIKEDPVTKVVTGLNAGKEYEFVVTYSYKEGN----SEIRAIYSNIAV      795
                    *:*:: :  .: :   . .**.:***: ..:*:***  .    .    .* : : **:..

00774               QRTNVNSSN-NNNNITGNGSNSTSGSSTGTTSIVVGNNNSTKTPTSLTLTLPGS-GY-ST      852
SFBNYU_003340       ATTKPGSSSSSGGTISGG---SSSSSSTGSTTVNVTSTNSTKGSTSISVTIPSGVSYTES      852
                      *: .**. ....*:*.   *:*.****:*:: * ..****  **:::*:*.. .* .:

00774               SYAPVVRGIKYKGTDGKVVTATSEQFANVKASYSSDGKSIILEGLVPDKDYTEISVDYTD      912
SFBNYU_003340       SANPTPVGFKYKDKNGKTVIETNDQYSNVKARFQN--GKVVLEGLVPGKEYTEISINYND      910
                    *  *.  *:***..:**.*  *.:*::**** :..   .::******.*:*****::*.*

00774               NNNKTRTLVIRDFKLTSVVDTLESYLRNVYTVVFDRPADEAGYNYHLNNLKSKKVSLRDF      972
SFBNYU_003340       KNNKTKTLVLKNVKIDSTS-EGQSYLANVYNVVFNRPADENGYMFHLGNLTSKKVSLRAF      969
                    :****:***:::.*: *.    :*** ***.***:***** ** :**.**.******* *

00774               LLNILNEKEFLGRYTTTEGKVEALYSAIVSRKSDEAGKAFWVNEYKTLLAVYGSEDNTLK      1032
SFBNYU_003340       LLNMINEKEFDKLYTTAENKVEALYNAIVNRDSDTQGKAFWVAEYKKLLSVYGSSSATLQ      1029
                    ***::*****   ***:*.******.***.*.**  ****** ***.**:****.. **:

00774               AIADRMVNENELKELANKLGVQW          1055
SFBNYU_003340       ALADRMVNENELKTLAQKLNFEW          1052
                    *:*********** **:**..:*
```

Supplementary Figure 7. Clustal W alignment of the amino acid sequences of gene 00774 from SFB-Human-IMAG and SFBNYU_003340. Turquoise labelling indicates the predicted signal peptide. Yellow labelling indicates the T_h17 stimulating epitopes from SFBNYU_003340 as described earlier (Yang et al. 2017). Residues labelled in green depicts conserved positions in the C-terminally located motifs CR-1A, CR-2A, CR-2A and CR-2B earlier identified in many tentatively extracellular SFB proteins (Pamp et al. 2012). Histidine (H), colored in grey indicates deviation from the consensus motif described by Pamp et al.

Supplementary tables

Parameter	Value
Completeness (%)	85.58
Contamination (%)	0
Genome size (bp)	1314549
Number of contigs	153
N50 contig length (bp)	11763
Mean contig length (bp)	8591
Longest contig (bp)	55026
GC (%)	26.98
Number of protein-coding genes	1276
Number of 16S rRNA genes	1
Number of 28S rRNA genes	1
Number of tRNA genes	28

Supplementary Table 1. Summary statistics for SFB-human-IMAG.

CAZyme	SFB-human-IMAG	Secretion signal
CE4	sfb.merged_00222	SP
CE4	sfb.merged_00371	SP
CE4	sfb.merged_00421	SP
CE4	sfb.merged_00862	-
CE4	sfb.merged_01243	SP
GH1	sfb.merged_00525	-
GH144	sfb.merged_00150	-
GH23	sfb.merged_00563	SP
GH23	sfb.merged_01271	-
GH3	sfb.merged_00630	
GH89	sfb.merged_00532	SP
GH94	sfb.merged_00096	-
GH94	sfb.merged_00149	-
GH94	sfb.merged_01168	-
GT2	sfb.merged_00325	-
GT2	sfb.merged_01223	-
GT2	sfb.merged_01225	-
GT26	sfb.merged_00218	-
GT28	sfb.merged_00181	-
GT28	sfb.merged_01254	-
GT4	sfb.merged_00220	-
GT4	sfb.merged_00863	-
GT4	sfb.merged_01184	-
GT4	sfb.merged_01185	-
GT4	sfb.merged_01277	-
GT51	sfb.merged_00191	-
GT51	sfb.merged_00331	-
GT84	sfb.merged_00096	-

Supplementary Table 2. Carbohydrate-active enzymes (CAZymes) in SFB-human-IMAG. CAZyme anotations were inferred using hmmscan against the dbCAN (http://bcb.unl.edu/dbCAN/) database. Reported hits all have e-value < 1e-18 and coverage > 0.35. Secretion signal was predicted with SignalP.
