# Supplementary material for: Genome sequence of segmented filamentous bacteria present in the human intestine
Source: Commun Biol. 2020 Sep 4;3:485. doi: 10.1038/s42003-020-01214-7 (PMC7474095; doi:10.1038/s42003-020-01214-7)
Supplement: Supplementary file 2 — Description of Additional Supplementary Files [file 42003_2020_1214_MOESM2_ESM.pdf]

Description of Additional Supplementary Files

File Name: **Supplementary Data 1**
Description SFB pangenome protein clusters
